# Efficacy of a Text-Based Mental Health Coaching App in Improving the Symptoms of Stress, Anxiety, and Depression: Randomized Controlled Trial

**DOI:** 10.2196/46458

**Published:** 2023-09-22

**Authors:** Yee Siew Lim, Jia Hui Quek, Xiu Wei Ching, Dominic Tao Ran Lim, Kean Ghee Lim, Chandramani Thuraisingham, Parikial Philip George

**Affiliations:** 1 International Medical University (IMU) Seremban Malaysia

**Keywords:** digital mental health, mobile health, randomized control trial, mobile phone, mental health, Depression, Anxiety, and Stress Scale–21 items, DASS-21, Asia, Malaysia

## Abstract

**Background:**

Stress, anxiety, and depression are major mental health concerns worldwide. A wide variety of digital mental health interventions have demonstrated efficacy in improving one’s mental health status, and digital interventions that involve some form of human involvement have been shown to demonstrate greater efficacy than self-guided digital interventions. Studies demonstrating the efficacy of digital mental health interventions within the Asian region are scarce.

**Objective:**

This study aimed to investigate the potential efficacy of the digital mental health intervention, ThoughtFullChat, which consists of one-on-one, asynchronous, text-based coaching with certified mental health professionals and self-guided tools, in improving self-reported symptoms of depression, anxiety, and stress. The study also aims to examine the potential differences in efficacy among occupational subgroups and between sexes.

**Methods:**

A randomized controlled study was conducted among housemen (trainee physicians), students, faculty members, and corporate staff at International Medical University, Malaysia. A total of 392 participants were enrolled and randomized to the intervention (n=197, 50.3%) and control (n=195, 49.7%) groups. Depression, anxiety, and stress symptoms were measured using the Depression, Anxiety, and Stress Scale–21 items at baseline and after the 3-month intervention period. The Satisfaction with Life Scale and Brief Resilience Scale were also included, along with a questionnaire about demographics.

**Results:**

Significant decrease was observed in depression (*P*=.02) and anxiety (*P*=.002) scores in the intervention group. A subgroup (corporate staff) also demonstrated significant decrease in stress (*P*=.005) alongside depression (*P*=.006) and anxiety (*P*=.002). Females showed significant improvements in depression (*P*=.02) and anxiety (*P*<.001) when compared with males.

**Conclusions:**

This study provides evidence that the ThoughtFullChat app is effective in improving the symptoms of depression, anxiety, and stress in individuals, particularly among corporate staff from the educational field. It also supports the notion that mobile mental health apps that connect users to mental health professionals in a discreet and cost-efficient manner can make important contributions to the improvement of mental health outcomes. The differential improvements among occupational subgroups and between sexes in this study indicate the need for future digital mental health app designs to consider an element of personalization focused on systemic components relating to occupation.

**Trial Registration:**

Clinicaltrials.gov NCT04944277; https://classic.clinicaltrials.gov/ct2/show/NCT04944277

## Introduction

### Background

Mental health is a subject of great concern in health care, with up to US $1 trillion each year spent on selected mental illnesses such as depression and anxiety worldwide [1] In the Asian region, pooled prevalence estimates from a meta-analysis study based in South Asia indicate depression and anxiety to reach >25% in large representative populations [2]**.** The countries studied were Afghanistan, Bangladesh, Bhutan, India, the Maldives, Nepal, Pakistan, and Sri Lanka.

According to the UK Office for National Statistics, the prevalence of moderate to severe depression in working adults increased from 5.5% to 7.5% and from 18% to 19.7% during the COVID-19 pandemic. In comparison, the depression rate among nonworking adults did not fluctuate significantly (24.8% before the pandemic to 26.6% during the pandemic) [3]**.** The Malaysian National Health and Morbidity 2015 survey found the prevalence of mental health problems among adults in Malaysia to be almost tripled from 11.2% in 2006 to 29.2% in 2015. Regarding depression, 1 in 5 cases were reported among Malaysian university students [4].

Odriozola-González et al [5] studied the mental well-being of Spanish university students from March 2020 to April 2020. Their results showed 34.19% to be experiencing moderate to extremely severe depression symptoms, 21.34% with severe anxiety symptoms, and 28.14% with moderate to extremely severe stress symptoms. Other important high-risk groups highlighted in the extant literature include health care workers [6,7] and academics [8-11]**.**

Most people experiencing mental health problems are unable to access treatment owing to a variety of reasons that include the lack of availability of services, time constraints, transportation problems, high costs, and stigma [2,12-14]. Digital mental health interventions, commonly defined as mental health interventions that use digital tools (ie, mobile apps or artificial intelligence–based software) to augment mental health care and improve its delivery, access, and effectiveness [15], have been known to be particularly useful in overcoming the barrier of stigma owing to increased privacy and anonymity [16]. Such interventions also improve access to preventive care [15], thus enabling a proactive approach to mental well-being. In terms of efficacy, digital mental health interventions have been shown to be as effective as traditional approaches [17] but without the typical barriers to accessibility that are linked to these latter approaches. Particularly in Asia, where the stigma associated with mental disorders can be considered “worse than the disease” [18], individuals may prefer digital interventions owing the level of anonymity and privacy afforded by them. Several digital mental health interventions have been proven to be effective [17,19], with evidence pointing to great effectiveness among interventions that combine a human therapist with digital assistance of some form, as compared with purely self-guided interventions [20].

Although many studies examine the efficacy of digital mental health interventions among different occupational groups such as university students [5], health care workers [6,8], physicians [7], and faculty staffs [10], very few emphasize the differential impact of these interventions among occupational groups or even between sexes. There is some evidence indicating that females are more likely to use and benefit from digital or web-based mental health interventions as compared with males [21,22]; however, these observations emphasize use rather than efficacy**.** Knowing the differential impact of digital mental health interventions among different occupational groups and sexes may provide useful insights that lead to more inclusive and effective intervention designs.

Most studies of digital mental health interventions are conducted in the West, with very little available data from Asia or other non-Western populations. Notably, 2 recent studies [23,24] in Singapore had highlighted the effectiveness of self-guided interventions in reducing stress and improving body image and self-compassion. To the best of our knowledge, there are no studies assessing the effectiveness of digital interventions that combine the services of a mental health professional and self-guided tools within an Asian sample at the time of writing. There are also no digital mental health studies in the region that emphasized improvements in symptoms of depression and anxiety.

In this study, we speculated that the ThoughtFullChat (TFC) mobile app, a one-on-one, asynchronous, text-based mental health coaching app equipped with self-guided tools, could potentially improve symptoms of depression, anxiety, and stress as measured by the Depression, Anxiety, and Stress Scale–21 items (DASS-21) scale. They also intended to explore the differences in efficacy among different subgroups based on occupation (medical housemen, corporate staff, undergraduate students, and faculty members) and sex, as this may present implications to mental health intervention design strategies among these different groups. The study was conducted on a Malaysian sample affiliated with the International Medical University (IMU), Malaysia, making it among the first to explore this subject in an Asian context.

### Objectives

This study examined the potential efficacy of the TFC mobile app among physicians in training (housemen), students, faculty members (academic staff), and corporate staff (non–health care) in reducing the symptoms of self-reported depression, anxiety, and stress. The study also examined the potential improvements in self-reported resilience and satisfaction with life after the intervention. Sex differences in improvements were analyzed, and feedback about user satisfaction was sought.

The first hypothesis that was proposed was that there would be improvement in depression, anxiety, and stress symptoms in the general sample and the subgroups after the intervention, consistent with previous observations in studies. The second hypothesis was that females would show more significant improvement than males, given the nature of the app that involved asynchronous texting with a mental health professional and other self-guided tools, which have been previously observed to be more engaging with females [22].

## Methods

### Study Design

This randomized controlled trial was conducted among housemen, university students, faculty members, and corporate staff at IMU from July 26, 2021, to October 26, 2021. The study was also registered on Clinicaltrials.gov (NCT04944277).

### Ethical Considerations

All procedures were approved by the research ethics committee of IMU on March 18, 2021 (CSc/Sem6[032021, CSc/Sem6[04]2021, CSc/Sem6[05]2021, CSc/Sem6[06]2021). CONSORT (Consolidated Standards of Reporting Trials) guidelines were followed, and the study was conducted in accordance with the Declaration of Helsinki (as revised in 2013).

This study adheres to rigorous ethical standards, encompassing human participant research ethics review, exemptions, and approvals; ensuring informed consent; protecting privacy and confidentiality; and affirming data anonymity or deidentification, while strictly refraining from providing any form of compensation for participation in the study.

Enrolled participants were assigned a code, which was used as the sole identifier during this study. The identity of the participants was not revealed to the mental health professionals who served them. The emergency contact details of each participant were requested and stored by ThoughtFull’s internal clinical team to be used in case of an emergency. The internal team had no access to the chat system or any communications between the mental health professional and participants. All participants were informed about the limits to privacy and the confidentiality policy before joining the study.

The information about the study was conveyed through an email and Microsoft Teams. By replying to the email, stating their agreement to participate in this study, and completing the registration process, participants demonstrated their informed consent to participate in the study.

### Participants

#### Enrollment

Participants were contacted through email and Microsoft Teams. A webinar on mental health resilience was conducted jointly by the ThoughtFull team and the clinical research team to introduce the research project and recruit participants. Interested participants were required to complete DASS-21, Satisfaction with Life Scale (SWLS), and Brief Resilience Scale (BRS). To maximize the response rate, a written notice and verbal reminder via phone were given to the nonresponding participants after the initial distribution of the questionnaires and before the commencement of the intervention.

#### Inclusion and Exclusion Criteria

The inclusion criteria include medical practitioners (housemen) who undergo internship training in any Malaysian hospital; are current IMU faculty members, students, and corporate staff of IMU who are competent in English; and are experiencing none to severe stress, anxiety, or depression based on their DASS-21 scores. All participants were aged >18 years. Participants who had extremely severe stress, anxiety, or depression were excluded from the study and were provided with helplines and contacts for mental health support services through the study information sheet. High scores were excluded owing to the nature and focus of the asynchronous text-based intervention, which is primarily preventive. More comprehensive and direct interventions may be required to mitigate potential harm that may be caused to those who have heightened symptoms. Hence, the intervention itself, being preventive in nature, is not meant for use by those who have severe symptoms. Moreover, precise cutoffs for the different levels of symptoms are provided in Table 1.

**Table 1:** *Depression, Anxiety, and Stress Scale–21* items scoring guide.

Levels	Depression (score), range	Anxiety (score), range	Stress (score), range
Normal	0-4	0-3	0-7
Mild	5-6	4-5	8-9
Moderate	7-10	6-7	10-12
Severe	11-13	8-9	13-16
Extremely severe	≥14	≥10	≥17

#### Sample Size Calculation

This interventional study with TFC involved the measurement of 5 variables: Depression, Anxiety, and Stress (DASS-21), Resilience and Satisfaction of Life scores. We used anxiety as the primary outcome for our sample size estimation, as previous analysis performed by ThoughtFull (ThoughtFull World, unpublished data, December 2020) showed that the most effective improvements were to be found with this construct.

The TFC app had an odds ratio change of 0.5 to 0.6, based on past data provided by ThoughtFull (ThoughtFull World, unpublished data, December 2020). Thus, an odds ratio of 0.54 was selected for use. This is based on individuals with anxiety moving into the normal range of scores or improving by 1 grade of severity. The sample size calculator obtained from OpenEpi [25] provided a sample size of 384, with 192 (50%) in each group. This study had 50.3% (197/392) of participants in the intervention group and 49.7% (195/392) in the control group, satisfying the sample size estimation.

### Randomization

Block randomization was chosen to allocate participants into intervention and control groups for our study. Before the beginning of the trial, we used Research Randomizer [26], a web-based software, to assign participants to intervention and control groups. We randomized 5 participants from every 10 respondents to our questionnaire. In total, 5 unique sets of numbers were generated, and the corresponding participants were allocated to the intervention group. The procedure was repeated for every 10 respondents.

### Treatments

This trial used a 1 × 1 factorial design (intervention group with free 3-month access to the coaching app vs control group with no access to the app). Participants in the control group were not introduced or given free access to the TFC app but were not restricted from using other apps or obtaining other forms of intervention during the study period.

#### Intervention Group

The intervention group was given a 3-month free access to the TFC app, a subscription-based mobile platform that empowers users to proactively engage with their mental health. The TFC app is a commercially available solution that is currently used as a coaching tool for the improvement of mental health outcomes in individuals with no formal diagnosis.

**Figure 1:**
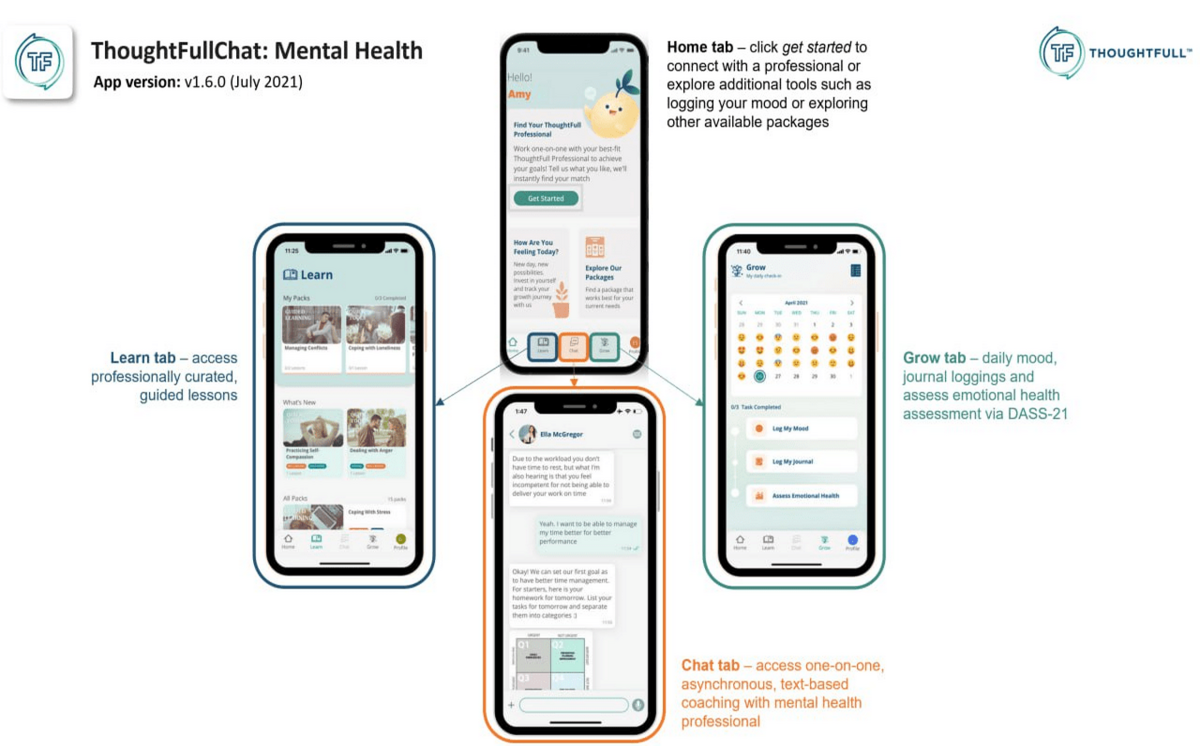
Screenshots of the ThoughtFullChat app. DASS-21: Depression, Anxiety, and Stress Scale–21 items.

Participants had full access to one-on-one, daily, asynchronous, text-based coaching with certified mental health professionals and self-guiding tools (ie, daily mood loggings, professionally curated guided lessons, and emotional health assessment using DASS-21). These mental health professionals had at least a master’s degree with ≥3 years of experience in working as counselors or clinical psychologists based in Singapore and Malaysia. Figure 1 shows the screenshots of the TFC app (version 1.6.0; latest version as of July 2021)—home tab, learn tab, chat tab, and grow tab.

#### Control Group

The control group was not given access to the TFC app; however, they were not barred from obtaining and using the app (or other apps) of their own accord. They were also provided with other nondigital resources such as helplines or counseling resources to be used in case of need.

Participants of both groups were assessed after 3 months of the intervention period, using a digital questionnaire comprising of all the instruments used (refer to the *Instruments* section).

### Instruments

The instruments used in the questionnaire were DASS-21, SWLS, and BRS.

#### DASS-21 Questionnaire

DASS-21 is a self-report scale used to screen for anxiety, depression, and stress. Each of the main scales contains 7 subscales, which sum up to 21 subscales. The sum scores for each scale (anxiety, depression, and stress) were categorized into normal, mild, moderate, severe, and extremely severe based on the severity ratings (Table 1) by Lovibond and Lovibond [27]. Previous studies have shown evidence for adequate validity and strong consistency (α=.90) of DASS-21 among the Malaysian student population [28].

Scores within the normal range indicate minimal or no depressive or anxiety or stress symptoms. Scores within the mild range indicate the presence of notable depressive or anxiety or stress symptoms that may be manageable without significant impairment. Scores within the moderate range indicate the presence of notable depressive or anxiety or stress symptoms that may be interfering with daily functioning. Scores within the severe range indicate the presence of severe depressive or anxiety or stress symptoms that may significantly affect daily functioning. Scores within the extremely severe range indicate the presence of severe depressive or anxiety or stress symptoms that may significantly affect daily functioning and require intervention. DASS-21 alone does not act as a tool for official diagnosis but is instead a screening tool.

#### SWLS Questionnaire

SWLS measures the individual’s subjective satisfaction with their overall life and consists of 5 items, with each using a 7-point Likert-type scale. This scale assesses different aspects of life satisfaction including alignment with ideals, life quality, general satisfaction, attainment of goals, and acceptance of past choices. The sum scores on each item were categorized into extremely satisfied (31-35), satisfied (26-30), slightly satisfied (21-25), neutral (20), slightly dissatisfied (15-19), dissatisfied (10-14), and extremely dissatisfied (5-9). SWLS has great validity and consistency (α=.86) among the clinical outpatients in Malaysia [29].

#### BRS Questionnaire

BRS measures the individual’s resilience, which is the ability to bounce back from adversity and cope with challenges. It consists of 6 items, with each using a 5-point Likert-type scale. This scale assesses different aspects of resilience including the ability to recover quickly, handle difficult situations, remain focused under pressure, and maintain an optimistic outlook. Scores of 6 items were summed together and divided by the number of questions answered. The score was interpreted based on the recommended range, which is low resilience (1-2.99), normal resilience (3-4.30), and high resilience (4.31-5). BRS had also been validated in the past and had shown high reliability (α=.93) among the Malaysian student population [30].

#### TFC App Feedback

Apart from measuring the symptoms of depression, anxiety, stress, resilience, and satisfaction with life, participants were also asked to indicate their general satisfaction with and perceived ease of use of the app.

### Pilot Study

A pilot study of the questionnaire was conducted among individuals who were demographically similar to the target population (n=20). These participants were not included in the study population. The aim of the pilot study was to ensure that the questions were understood and perceived correctly. The pilot study found that all participants (20/20, 100%) understood the context well and could answer the questionnaires without any obstructions.

### Statistical Analysis

The collected data were exported from Microsoft Forms to Excel Sheets and analyzed using the IBM SPSS version 28.0.0.0. Descriptive statistics were calculated for the participant’s sociodemographic factors and user experience of the app in the intervention group. The mean and SD for the DASS-21, SWLS, and BRS scores before and after the intervention are presented. Wilcoxon signed rank test was used to calculate the significant differences in the mean of the 3 metrics (DASS-21, SWLS, and BRS) of the 2 groups. *P* value <.05 is considered as statistically significant. Rank-biserial correlation value was used to calculate the effect size of each result.

## Results

### Overview

**Figure 2:**
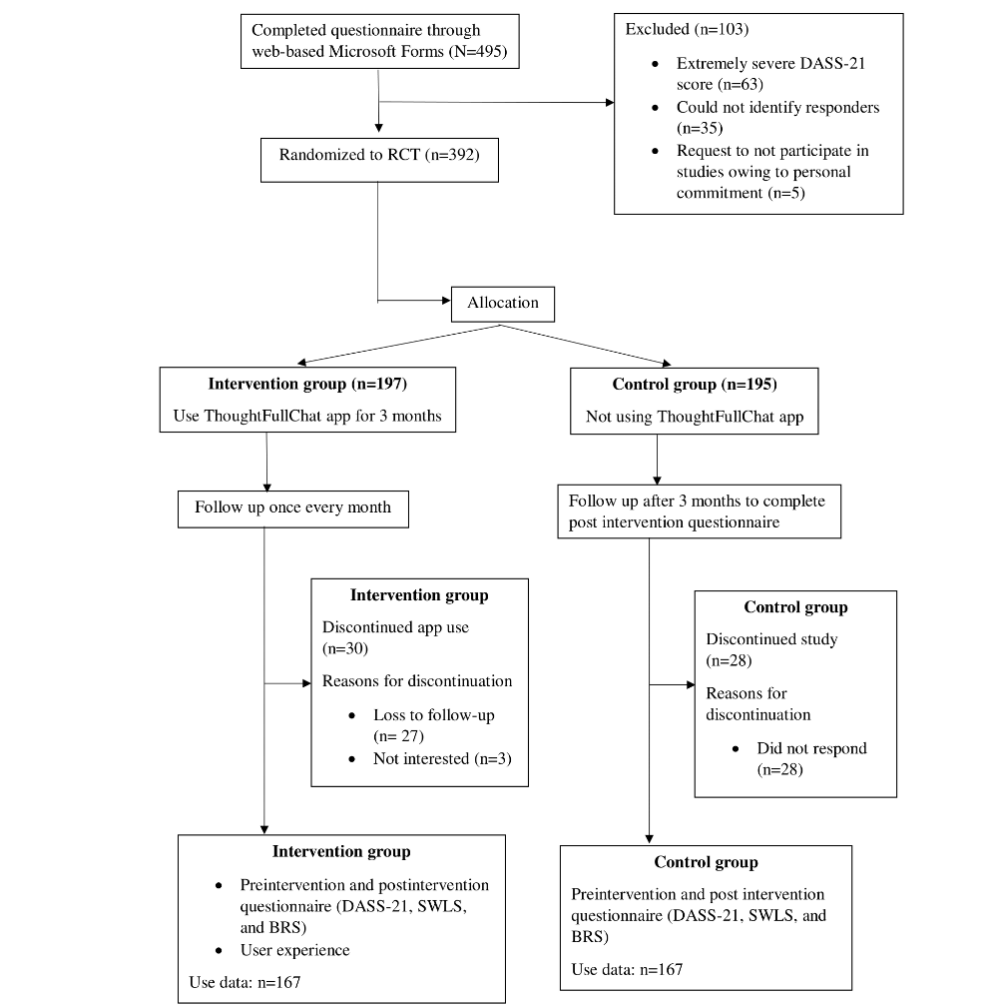
CONSORT (Consolidated Standards of Reporting Trials) diagram showing the selection process of this study. BRS: Brief Resilience Scale; DASS-21: Depression, Anxiety, and Stress Scale–21 items; RCT: randomized controlled trial; SWLS: Satisfaction with Life Scale.

Figure 2 explains the flow of this study. There were 495 individuals who responded to our preinterventional questionnaire. Upon screening the participants’ DASS-21 scores, 79.2% (392/495) were enrolled and randomized to the intervention and control groups. The remaining 12.7% (63/495) of the respondents did not meet the inclusion criteria owing to severe DASS-21 scores (refer to the cutoff scores for severe DASS-21 in Table 1). We excluded 0.1% (35/495) of the respondents owing to incomplete questionnaires. During the study, 15.2% (30/197) of the participants from the intervention group requested to be removed from participating. In the control group, 14.4% (28/195) of the participants did not complete the follow-up questionnaire. After 3 months, there were 167 participants in each group who completed the intervention.

### Sociodemographic Descriptions

Sociodemographic characteristics of the respondents are presented in Table 2. The sample was mostly females (215/334, 64.2%), those aged between 18 and 29 years (178/334, 53.3%), and those who are single (194/334, 58%). The respondents’ nationality is separated into 2 major categories: Malaysian (304/334, 91%) and others (30/334, 9%). Most of our respondents have a high level of education, with bachelor’s degree (192/334, 57.5%) and master’s degree (67/334, 20%). In terms of income status, most of them (178/334, 53.3%) are in the middle 40% category. We categorized the respondents into bottom 40% (<Ringgit Malaysia [RM] 4849 [US $1047.53]), middle 40% (RM 4850 [US $1047.53] to RM 10,959 [US $2367.47]), and top 20% (>RM 10,959 [US $2367.47]) of the household income classification in Malaysia by following the income range set by the Department of Statistics, Malaysia, in 2019.

**Table 2:** Descriptive statistics about the sociodemographics of participants (N=334).

Characteristics			Intervention group (n=167), n (%)		Control group (n=167), n (%)
**Age group (years)**					
	18-29	98 (58.7)		80 (47.9)	
	30-49	50 (29.9)		71 (42.5)	
	>50	19 (11.4)		16 (9.6)	
**Sex**					
	Male	52 (31.1)		67 (40.1)	
	Female	115 (68.9)		100 (59.9)	
**Nationality**					
	Malaysian	151 (90.4)		153 (91.6)	
	Non-Malaysian	16 (9.6)		14 (8.4)	
**Highest level of formal education**					
	Secondary school	2 (1.2)		6 (3.6)	
	Preuniversity	19 (11.4)		24 (14.4)	
	Diploma	1 (0.6)		2 (1.2)	
	Bachelor’s degree	93 (55.7)		99 (59.3)	
	Master’s degree	40 (23.9)		27 (16.2)	
	PhD	12 (7.2)		9 (5.4)	
**Family income, RM^a^ (US $)**					
	<4360 (B40^b^; $2367.47)	35 (20.9)		35 (20.9)	
	4360 to 9619 (M40^c^; $2367.47 to $2367.47)	84 (50.3)		94 (56.3)	
	>9619 (T20^d^; $2367.47)	46 (27.5)		36 (21.6)	
	Not disclosed	2 (1.2)		2 (1.2)	
**Relationship status**					
	Single	99 (59.3)		95 (56.9)	
	Married	66 (39.5)		71 (42.5)	
	Others	2 (1.2)		1 (0.6)	

^a^RM: Ringgit Malaysia.

^b^B40: Bottom 40% of the household income classification in Malaysia.

^c^M40: Middle 40% of the household income classification in Malaysia.

^d^T20: Top 20% of the household income classification in Malaysia.

### Overall Outcomes

Decrease in overall mean scores of depression, anxiety, and stress was observed in the intervention group, whereas the mean scores were on the rise in the control group (Table 3**)**. The decrease in depression (*P*=.02) and anxiety (*P*=.002) in the intervention group was significant. Changes in the SWLS and BRS scores showed no significant difference.

DASS-21, SWLS, and BRS scores of the participants in each subgroup are shown in Table 4. The overall effect size of the subgroups was generally small, except for the corporate staff group, which had a moderate effect size (Rank-biserial correlation=0.60). SWLS and BRS scores were not significant for all groups, except corporate staff.

**Table 3:** Comparing means, *P* values, and effect sizes for outcome variables between overall intervention and control groups before and after the 3-month study period.

Outcome variable	Intervention group					Control group			
	Preintervention score, mean (SD)	Postintervention score, mean (SD)	*P* value	Rank-biserial correlation	Preintervention score, mean (SD)		Postintervention score, mean (SD)	*P* value	Rank-biserial correlation
Depression	3.49 (3.30)	2.90 (3.52)	.02	0.244	3.37 (3.18)		3.99 (5.12)	.31	−0.103
Anxiety	3.39 (2.60)	2.79 (3.26)	.002	0.314	3.19 (2.62)		3.68 (4.69)	.70	−0.039
Stress	5.30 (3.65)	5.02 (4.50)	.29	0.102	4.85 (3.49)		5.20 (5.21)	.10	−0.0006
Satisfaction with Life	24.36 (5.87)	24.61 (6.40)	.22	−0.118	23.79 (5.71)		24.23 (5.73)	.29	−0.10
Resilience	3.58 (3.21)	3.35 (0.62)	.13	0.150	3.32 (0.58)		3.34 (0.61)	.67	0.044

**Table 4:** Comparing the means, *P* values, and effect sizes between each subgroup.

					Intervention group													Control group									
					Preintervention score, mean (SD)			Postintervention score, mean (SD)			*P* value			Rank-biserial correlation			Preintervention score, mean (SD)				Postintervention score, mean (SD)			*P* value			Rank-biserial correlation
**Housemen group (n=91)**																											
	Depression			3.65 (3.33)			3.58 (3.67)			.57			0.107			3.54 (3.21)				4.88 (3.87)			.06			−0.358	
	Anxiety			3.88 (2.57)			3.77 (4.20)			.53			0.113			2.95 (2.51)				3.91 (3.79)			.16			−0.280	
	Stress			5.46 (4.77)			5.42 (4.77)			.97			0.007			4.98 (3.58)				5.56 (3.89)			.50			−0.123	
	Satisfaction with Life			21.75 (5.79)			22.15 (4.88)			.79			−0.049			20.88 (6.04)				22.19 (5.19)			.17			−0.262	
	Resilience			3.02 (0.57)			2.96 (0.42)			.40			0.168			3.07 (0.51)				3.12 (0.65)			.61			−0.107	
**Student group (n=73)**																											
	Depression			3.63 (3.57)			2.84 (3.45)			.37			0.176			4.63 (3.21)				4.94 (4.93)			.71			0.076	
	Anxiety			3.03 (2.47)			2.55 (2.33)			.01			0.355			3.25 (2.85)				3.58 (3.83)			.70			−0.086	
	Stress			4.89 (3.49)			4.79 (3.57)			.86			0.034			5.83 (3.25)				5.25 (4.21)			.35			0.189	
	Satisfaction with Life			24.66 (5.82)			22.45 (7.47)			.04			0.389			22.70 (5.84)				23.42 (7.14)			.58			−0.117	
	Resilience			3.37 (0.89)			3.12 (0.68)			.08			0.346			3.36 (0.64)				3.20 (0.68)			.17			0.302	
**Faculty group (n=57)**																											
	Depression			3.78 (3.24)			3.17 (4.07)			.21			0.234			2.50 (3.05)				4.70 (10.2)			.66			−0.165	
	Anxiety			3.25 (2.73)			2.56 (3.05)			.24			0.259			2.26 (2.42)				4.80 (9.57)			.37			−0.308	
	Stress			6.61 (4.49)			6.78 (5.62)			.88			−0.032			5.25 (4.51)				8.90 (10.55)			.20			−0.345	
	Satisfaction with Life			27.08 (4.94)			27.39 (6.87)			.23			−0.280			28.05 (4.21)				26.93 (5.96)			.74			0.094	
	Resilience			4.94 (6.71)			3.81 (0.51)			.60			0.115			3.75 (0.64)				3.85 (0.57)			.42			−0.222	
**Corporate staff (n=113)**																											
	Depression			2.90 (3.14)			1.90 (2.74)			.006			0.566			2.80 (3.02)				2.70 (3.26)			.93			0.015	
	Anxiety			3.30 (2.63)			2.10 (2.77)			.002			0.597			3.60 (2.58)				3.30 (3.36)			.14			0.227	
	Stress			4.50 (3.55)			3.40 (3.29)			.005			0.564			4.10 (3.13)				3.80 (3.30)			.44			0.120	
	Satisfaction with Life			24.70 (5.70)			27.10 (4.75)			<.001			−0.618			24.90 (4.70)				25.20 (4.65)			.91			−0.017	
	Resilience			3.60 (0.69)			3.60 (0.49)			.68			−0.080			3.47 (0.61)				3.40 (0.45)			.46			0.118	
**Age group**																											
	**18-29 years**																										
		Depression	3.79 (3.47)			3.26 (3.55)			.13			0.190			3.95 (3.22)				4.74 (4.09)			.24			−0.160		
		Anxiety	3.63 (2.57)			3.24 (3.51)			.06			0.242			3.09 (2.63)				3.80 (3.71)			.16			−0.205		
		Stress	5.33 (3.31)			5.03 (4.18)			.44			0.097			5.30 (3.52)				5.40 (3.95)			.93			0.013		
		Satisfaction with Life	23.1 (5.90)			22.66 (6.04)			.49			0.085			21.94 (5.90)				22.63 (5.97)			.34			−0.133		
		Resilience	3.26 (0.75)			3.08 (0.57)			.05			0.254			3.19 (0.58)				3.12 (0.61)			.41			0.126		
	**>30 years**																										
		Depression	3.03 (3.02)			2.36 (3.41)			.04			0.343			2.83 (3.07)				3.31 (5.86)			.84			−0.031		
		Anxiety	3.04 (2.61)			2.14 (2.76)			.01			0.413			3.29 (2.63)				3.57 (5.46)			.38			0.125		
		Stress	5.26 (4.10)			5.01 (4.95)			.49			0.107			4.44 (3.43)				5.01 (6.16)			.90			0.018		
		Satisfaction with Life	26.14 (5.37)			27.38 (5.88)			.002			−0.461			25.51 (4.97)				25.77 (5.05)			.71			−0.048		
		Resilience	4.28 (4.89)			3.72 (0.49)			.91			−0.018			3.49 (0.62)				3.54 (0.54)			.93			−0.012		
**Sex**																											
	**Male**																										
		Depression	3.56 (3.57)			3.33 (3.88)			.32			0.178			3.37 (3.27)				3.69 (5.69)			.80			0.041		
		Anxiety	3.13 (2.54)			2.83 (3.09)			.53			0.105			3.03 (2.56)				3.39 (5.65)			.46			0.115		
		Stress	4.77 (3.38)			4.79 (4.12)			.95			0.012			4.54 (3.48)				4.70 (5.91)			.39			0.128		
		Satisfaction with Life	23.94 (6.78)			23.17 (7.05)			.77			0.156			24.43 (5.54)				24.51 (5.52)			.77			−0.019		
		Resilience	4.12 (5.67)			3.31 (0.60)			.30			0.347			3.40 (0.59)				3.48 (0.53)			.30			−0.018		
	**Female**																										
		Depression	3.43 (3.19)			2.71 (3.33)			.02			0.272			3.36 (3.14)				4.20 (4.73)			.13			−0.198		
		Anxiety	3.50 (2.62)			2.77 (3.35)			<.001			0.409			3.30 (2.67)				3.88 (3.94)			.26			−0.151		
		Stress	5.54 (3.75)			5.13 (4.68)			.24			0.137			5.06 (3.50)				5.53 (4.77)			.54			−0.076		
		Satisfaction with Life	24.55 (5.43)			25.26 (6)			.03			−0.249			23.35 (5.80)				24.09 (5.88)			.22			−0.151		
		Resilience	3.34 (0.73)			3.36 (0.64)			.69			0.048			3.26 (0.65)				3.25 (0.65)			.58			0.073		

### Housemen Group

The intervention group showed improvement in DASS-21 scores, but the results were not statistically significant. The control group showed a slight increase in DASS-21 scores, but this was also not statistically significant.

### Medical Students Group

The depression, anxiety, and stress levels decreased among the medical students in the intervention group, but the decrease was not statistically significant. However, the control group showed a slight increase in depression and anxiety but was not significant.

### Faculty Group

The depression and anxiety levels had shown some improvement in the intervention group but worsened in the control group. However, the differences were not significant.

### Corporate Staff Group

The intervention group had shown significant improvements in the DASS-21 scores (depression: *P*=.006; anxiety: *P*=.002; and stress: *P*=.005) as compared with the control group. It also showed significant improvement in the SWLS scores (*P*<.001).

### Age Group

This study found that the young group (aged 18-29 years) had high baseline levels of depression, anxiety, and stress compared with those aged >30 years. Significant improvement in depression (*P*=.04) and anxiety (*P*=.01) was seen in the group of individuals aged >30 years. In contrast, both age groups in the control group showed decrease in depression, stress, and anxiety.

### Sex

In the intervention group, depression and anxiety scores reduced for both females and males, but females showed significant improvements in depression (*P*=.02) and anxiety (*P*<.001). Females also showed significant improvements in SWLS scores. The changes in the control group were all nonsignificant.

### User Experience With the TFC App

Regarding user experience of the app, approximately 68.3% (114/167) of the users reviewed the app favorably, with 61.2% (102/167) saying that they will recommend the app to others and 74% (69/93) claiming that the app was easy to use (Table 5).

**Table 5:** User experience of the ThoughtFullChat app.

Item and response options			Frequency, n (%)
**How likely would you recommend ThoughtFullChat app to a friend or colleague? (n=167)**			
	Not at all	7 (4.2)	
	Somewhat unlikely	13 (7.8)	
	Neither likely nor unlikely	45 (26.9)	
	Somewhat likely	72 (43.1)	
	Very likely	30 (17.9)	
**Review of ThoughtFullChat app (n=167)**			
	Very unsatisfied	6 (3.6)	
	Unsatisfied	6 (3.6)	
	Neutral	41 (24.6)	
	Satisfied	89 (53.3)	
	Very satisfied	25 (14.9)	
**User-friendliness of ThoughtFullChat app (n=93; missing data: n=74)**			
	Very difficult to use	1 (1.1)	
	Difficult to use	3 (3.2)	
	Neither easy nor difficult to use	20 (21.5)	
	Easy to use	47 (50.5)	
	Very easy to use	22 (23.7)	

## Discussion

### Principal Findings

The goal of this study was to examine the potential effectiveness of a text-based mental health coaching app in improving mental well-being among adults without any known diagnosis of mental health problems. The results demonstrated partial support for the first hypothesis, that the use of the TFC app would result in reduced DASS-21 scores. There was significant decrease in scores of anxiety and depression in the overall sample, but the decrease in stress was not significant, possibly indicating a pathway to the reduction of depression and anxiety that is independent of stress scores. The analysis of occupational subgroups seemed to indicate great effectiveness of the intervention for corporate staff as opposed to the other subgroups. It is likely that the nature of the workplace environment (ie, a fixed schedule and with arguably great predictability as compared with students and housemen) may have contributed independently to the increased effectiveness observed. However, further studies, particularly of qualitative nature, may be required to further confirm this speculation. It is also worth noting that even in subgroups in which there were no significant improvements in the intervention group, deterioration was observed in the control group, likely indicating resistance to deterioration in the intervention group. This is a reasonable conjecture that requires much more in-depth analysis to be confirmed. This study highlights the importance of considering the type of occupation of the target group when choosing digital interventions as a strategy for mental health. An understanding of the differential pathways that led to improvement (ie, how the different occupational groups interacted with the app) could provide essential clues for the development of more effective interventions that are more inclusive and accommodative of occupational differences.

The second hypothesis, that females would show more significant improvements than males, was also supported by the results. Smail-Crevier et al [21] reported differential likelihood of use of e–mental health programs between females and males. Females preferred features relating to access of information, interactive self-help, well-being exercises, and text-based communications with mental health professionals, whereas males preferred a more gamified system. The TFC app in its current form seemed to match the first, with text-based communication being the primary focus of the intervention. The design and characteristics of a mental health coaching app are crucial in improving the user experience and thus mental health status. This study supports the notion that disparities in app design may unintentionally result in unequal access between the sexes. Improvements to the design that include a more gamified approach may be relevant in engaging males through the app; however, it is important to ensure that the app does not become addictive and thus counterproductive.

Taken together, the differential improvements among occupational subgroups and between sexes in this study indicate the need for future digital mental health app designs to consider an element of personalization. Although such personalization has often been suggested to include the use of individual psychological patterns and physiological markers [31], large systemic factors such as type of occupation or workplace environment may also prove to be essential. Although it is true that there is no one-size-fits-all approach, consideration of a variety of individual and systemic factors may lead to intervention designs that are more inclusive of and effective for a wide target.

In general, this study is consistent with previous studies in showing that digital mental health coaching apps can help improve mental health [32]. As the participants of this project belonged to a wide range of age groups and had different occupational backgrounds, the result of this study suggests that the app can be implemented for managing depression, stress, and anxiety symptoms for a diverse group of individuals in the general population, although with somewhat different levels of efficacy. The study also contributes to the limited baseline data available about digital mental health interventions in the Asian region.

### Strengths and Limitations

Digital mental health apps are becoming more widely available, and studies of these apps are largely conducted in Western populations. This study is among the few studies of digital mental health apps in Asia, thus adding to the limited but growing knowledge base on this subject within the region.

Although personalization in digital mental health care is a common direction, this study advocates for the consideration of large systemic factors such as type of occupation and sex when considering the personalization of mental health care. Keeping in mind that different subgroups within a population respond differently to a uniform intervention and understanding how these differences in response emerge through the interaction of systemic factors is likely to provide useful information that could guide efforts toward effective personalization of intervention design. This study presents preliminary evidence of such differences and represents a first step in this direction within an Asian context.

Limitations of the study include the absence of a blinding method. Participants who were assigned to the control group were aware that they will not be using the app. However, all participants from both groups were addressed using the same method, which was via email and SMS text messages. It is also important to note that the questionnaires used in this study are screening tools and not meant to confer any formal diagnosis. The study was therefore focused on prediagnosed individuals. Studies involving diagnosed individuals may provide more useful insights.

In addition, it is noted that the sample consists only of individuals associated with IMU and, therefore, may be subject to a certain uniformity of experience relating to their occupational environment. However, the housemen affiliated with IMU were still subjected to a wide range of experiences pertaining to their physical location, as they were based in various hospitals within the country. Future studies may consider using a wide range of participants from different organizations to be inclusive of a more diverse set of occupational experiences. Furthermore, the participants of this study were allowed to use the app with flexibility, and there was insufficient monitoring of their DASS-21 scores along with app use. Hence, there was insufficient control for other factors that may have affected DASS-21 scores. This includes the presence or absence of other stress factors such as working and family environments, which may have affected the results. Finally, no direct connections were made between using digital mental health apps and the barrier of stigma and perceived accessibility and acceptance of mental health interventions. This is perhaps a helpful area for further study within the landscape.

### Conclusions

This study provides evidence that the TFC app is effective in improving symptoms of depression, anxiety, and stress in individuals, particularly among corporate staff. It also demonstrated great improvements in females as compared with males, likely attributed to the design of the app. This is indicative of the need for a consideration of sex differences and occupational groups while designing digital mental health intervention strategies. Results also demonstrated positive user experience among participants, suggesting that similar digital tools can be implemented as an approach to tackling mental health issues within the Asian region.

## Appendix

Multimedia Appendix 1 CONSORT-eHEALTH checklist (V 1.6.1).

## Abbreviations

BRS: Brief Resilience Scale
CONSORT: Consolidated Standards of Reporting Trials
DASS-21: Depression, Anxiety, and Stress Scale–21 items
IMU: International Medical University
RM: Ringgit Malaysia
SWLS: Satisfaction with Life Scale
TFC: ThoughtFullChat

## Data Availability

The data sets generated and analyzed during this study are not publicly available owing to internal data handling policies pertaining to privacy and confidentiality but are available from the corresponding author upon reasonable request.
